# Selenium-Enriched Yeast Relieves Hexavalent Chromium Toxicity by Inhibiting NF-κB Signaling Pathway in Broiler Spleens

**DOI:** 10.3390/ani12020146

**Published:** 2022-01-08

**Authors:** Yanbing Zhao, Dezheng Hao, Huan Zhang, Jingqiu Wang, Ci Liu

**Affiliations:** College of Veterinary Medicine, Shanxi Agricultural University, Jinzhong 030801, China; zhaoyanbing010@163.com (Y.Z.); haodezheng1998@163.com (D.H.); W13072626931@163.com (H.Z.); wjingq@163.com (J.W.)

**Keywords:** broiler spleen, hexavalent chromium, inflammation, NF-κB signaling pathway, selenium-enriched yeast

## Abstract

**Simple Summary:**

Hexavalent chromium is a common environmental pollution. It has been reported that hexavalent chromium threatens the health of humans and animals, so it is necessary to develop new, effective mitigation methods. Selenium is an indispensable micronutrient recently shown to be able to resist the toxicity of heavy metals. Selenium-enriched yeast has a high content of total selenium, which has the advantages of a high absorption rate and safety. Potassium dichromate and selenium-enriched yeast were used to construct single hexavalent chromium and combined selenium/hexavalent-chromium-exposed broiler models. Additionally, the ability to relieve the hexavalent chromium toxicity of selenium along with the molecular mechanisms focusing on inflammation induced by the NF-κB signaling pathway was investigated in this study. Histopathological assessment, serum biochemical tests, oxidative stress kits, enzyme-linked immunosorbent assay, quantitative real-time PCR, and Western blotting were used to detect indicators. We found that the oxidative stress induced by hexavalent chromium triggers NF-κB pathway-driven inflammatory responses in the broiler spleen and further reduces the immune function of broilers. Selenium-enriched yeast protects the spleen from the toxicity of hexavalent chromium exposure through inhibiting the NF-κB signaling pathway.

**Abstract:**

This study was conducted to investigate the molecular mechanisms of selenium (Se) antagonism of hexavalent chromium (Cr^6+^)-induced toxicity. Potassium dichromate (K_2_Cr_2_O_7_) and selenium-enriched yeast (SeY) were used to construct the single Cr^6+^ and combined Se/Cr^6+^ exposure broiler models, and then the broilers were randomly divided into four groups (C group, Se group, Se/Cr^6+^ group, and Cr^6+ ^group). After a 42-day experiment, the spleen tissues of broilers were excised and weighted. The antagonistic mechanisms of Se and Cr^6+^ were evaluated using histopathological assessment, serum biochemical tests, oxidative stress kits, ELISA, qPCR, and Western blotting. On the whole, there were no significant changes between the C and Se groups. The spleen organ index in the Cr^6+^ group was significantly decreased, but SeY increased spleen organ index to a certain extent. The levels of SOD and GSH were reduced, and the MDA content was elevated by Cr^6+^; however, these changes were mitigated by Se/Cr^6+^ exposure. Importantly, Cr^6+^ exposure induced a series of histopathological injuries in broiler spleen tissues, while these symptoms were significantly relieved in the Se/Cr^6+^group. Furthermore, Cr^6+^ significantly decreased the levels of T-globulin, IgA, IgM, and IgG in serum. Contrarily, dramatically more T-globulin IgA, IgM, and IgG were found in the Se/Cr^6+^group than in the Cr^6+^ group. Revealed by the results of qPCR and WB, the expressions of NF-κB, IκBα, and p-IκBα were upregulated in Cr^6+^ groups, while they were downregulated in Se/Cr^6+^ group compared to that in Cr^6+^ group. Besides IFN-γ and IL-2, the expressions of pro-inflammatory cytokines were significantly increased by Cr^6+^ exposure, but the SeY supplement relived the expression levels mediated by Cr^6+^ exposure. In conclusion, our findings suggest SeY has biological activity that can protect broiler spleens from immunosuppression and inflammation induced by Cr^6+^, and we speculate that the NF-κB signaling pathway is one of its mechanisms.

## 1. Introduction

Chromium (Cr) is commonly used as a micronutrient and a dietary supplement. The appropriate addition of Cr to the broiler diet can improve production performance and carcass quality and immunity, and can alleviate stress [1]. In nature, Cr exists in different valence forms, including Cr^0^, Cr^+^, Cr^3+,^ and Cr^6+^; however, Cr^6+^ is considered a ubiquitous environmental pollutant [2]. Multifold industrial operations have raised Cr^6+^ content in the environment, contaminating the ecological environment [3], ultimately affecting human and animal health through the food chain. As previously reported, Cr^6+^ is highly toxic and harmful to the digestive system, immune system, cardiovascular system, and other systems in the body [1,4,5,6].

Cr^6+^ mainly induces oxidative stress via promoting the formation of reactive oxygen species (ROS) in different tissues and organs [7]. Scholars discovered that oxidative stress induced by ROS was closely related to multiple damage responses [8]. In addition, inflammation was reported as an important toxicity mechanism of multiples heavy metals [9,10]. Inflammation is a defense response of the organism to stimulation, but excessive inflammation is harmful to animals. As a transcription factor, nuclear factor-κB (NF-κB) mediates cell growth, development, immune function, and other biological processes, dominated by involvement in inflammatory responses [11]. The activation of the NF-κB signaling pathway induces the expressions of such inflammatory genes as tumor necrosis factor-α (TNF-α), interleukin1β (IL-1β), nitric oxide synthase (iNOS), and cyclooxygenase-2 (COX-2), which leads to an inflammatory response [12,13]. The experiment of Yang et al. confirmed that copper could trigger oxidative stress to activate the NF-κB signaling pathway and further regulate the expression of TNF-α, IFN-γ, IL-1, IL-1β, IL-2, iNOS, and COX-2 in the chicken spleen [14]. Recently, a study showed that Cr^6+^ induced inflammatory responses by significantly increasing TNF-α levels in chicken hearts; at the same time, it damaged mitochondria and caused cell autophagy [15]. Therefore, the inflammation induced by the NF-κB signaling pathway is one of the mechanisms for studying Cr^6+^ poisoning.

The proteins in the blood are mainly composed of albumin and globulin, and serum globulin function mainly plays a role in anti-infection and anti-inflammatory [16]. Serum globulins are mainly composed of serum immunoglobulins. Immunoglobulins are important manifestations of humoral immunity. Spleen, as the largest peripheral immune organ in poultry, is an important organ for dominating immune responses and can produce immunoglobulins, such as immunoglobulin A (IgA), immunoglobulin G (IgG), and immunoglobulin M (IgM) [17]. The immunoglobulins play an important role in neutralizing toxins, bacteria, or viruses, regulating and activating complement, and they are also important indicators for immune function [18]. However, the spleen is likely to be affected by toxicity resulting from such heavy metals as cadmium, lead, and arsenic [19]. Wang et al. found that lead exposure could cause oxidative stress and inflammation in Japanese quail immune organs, manifested as lymphopenia and decreased serum IgM and IgG levels, thereby reducing immune function [20]. Adding fluoride to the feed can reduce the contents of IgA, IgG, and IgM in broilers, thereby reducing the immune function of broilers [21]. At the same time, we found that broilers showed a poor mental state, growth retardation, and dull and sparse feathers after Cr^6+^ exposure in this experiment. Hence, we speculated that reducing immune function is one of the toxic effects of Cr^6+^.

As an irreplaceable micronutrient, Selenium (Se) is one of the constituents of glutathione peroxidase (Gpx). Gpx is an enzyme that can resist oxidative stress and maintain the redox balance [22]. Se, as a food additive, mainly exists in two forms, including inorganic Se and organic Se (OS) [23]. Compared to inorganic Se, OS has higher absorption and utilization rates and is safer [24]. Recently, the application of Se-enriched yeast (SeY), a source of OS, as feed additives to keep healthy poultry and elevate Se level in carcass meat and eggs, has obtained approval and has been accepted [25]. Se can chelate a variety of heavy metals and presents the potential to hinder heavy-metal-trigged toxicity. For example, Se antagonizes Pb toxicity to inhibit the lymphocyte apoptosis in the chicken spleen [26]; and Se also helps the spleen eschew from HgCl_2_-triggered injury in chicken via controlling oxidative stress, inflammation, and apoptosis [27].

The specific mechanism developed by Se to efficaciously antagonize Cr^6+^ is inexplicit in broiler spleen tissues. In this study, we used potassium dichromate (K_2_Cr_2_O_7_) and SeY to establish the single Cr^6+^ and combined Se/Cr^6+^-exposed broiler model and investigated whether Se could alleviate immunosuppression and inflammation by the NF-κB signaling pathway in broilers exposed to Cr^6+^. This study enriches the theoretical mechanism for the damage caused by Cr^6+^ and its treatment methods.

## 2. Materials and Methods

### 2.1. Animal Experiment

The Animal Ethics Committee of Shanxi Agricultural University gave approval to all animal assays. We purchased 100 1-day-old broilers from Taigu Qingmu Breeding Co., Ltd. (Jinzhong, Shanxi, China) and raised them at the Experimental Animal Management Center of Shanxi Agricultural University. All animals experienced a week of adaptive period and were provided with sufficient water and feed. Then, the broilers were stochastically assigned into control group (C), SeY group (Se), SeY+Cr^6+^ group (Se/Cr^6+^), and Cr^6+^ group (Cr^6+^). There were 5 replicates per group, 5 broilers/replicates. According to previous studies, broilers received exposure of potassium dichromate (K_2_Cr_2_O_7_) at a dose of 0.037 g/kg·BW (8% LD_50_) [28,29], and we used SeY to feed broilers at 0.30 mg/kg [30,31]. We calculated the dose of K_2_Cr_2_O_7_ and added it to the distilled water with the daily weight gain of broiler. Broilers in C group were provided a basal diet and distilled water; in Se group, an additional 0.30 mg/kg of SeY was added to the basal diet; in Se/Cr^6+^ group, 0.30 mg/kg of SeY was added to the basal diet, and 0.037 g/kg·BW of K_2_Cr_2_O_7_ was added to the distilled water; and in Cr^6+^ group, we added 0.037 g/kg·BW of K_2_Cr_2_O_7_ to distilled water. The dosage of K_2_Cr_2_O_7_ was adjusted as the body weight of broilers increased. After 42 days, the broilers were fasted for 12 h while ensuring adequate drinking water, and then we recorded broiler weight. The broilers were sacrificed with sodium pentobarbital. We collected broiler blood and obtained serum after centrifugation, and detected relevant biochemical indicators. Then, the broilers were sacrificed, and the spleen tissues were immediately excised, washed with 0.9% saline, and weighed. One part was used for histopathological examination (fixed with 4% paraformaldehyde), and the other part was used for molecular biology analysis (stored at −80 °C).

### 2.2. Determination of the Organ Index

At the end of the experiment, the broiler weights and the spleen weights from all four groups were weighed and recorded. The organ index of the spleen was calculated by the following formula:

organ index = organ weight (g)/broiler weight (kg).

### 2.3. Histopathological Observation of Broiler Spleens

After removing spleen tissue blocks from 4% paraformaldehyde, we embedded the tissue blocks in paraffin and then dried the paraffin block completely. The microtome was used to cut the paraffin tissue blocks with a thickness of 3 μm and then put them on glass slides. Finally, we observed the morphological changes of the spleen tissues under an optical microscope (Nikon Eclipse E100, Nikon Co, Tokyo, Japan).

### 2.4. Determination of Oxidative Damage in the Spleen Tissues of Broilers

The spleen tissues were made into a homogenate and centrifuged, and the supernatant was collected. We measured the protein concentration by the BCA method, after which we used the corresponding kits (Nanjing Jiancheng Bioengineering Institute, Nanjing, China) to detect superoxide dismutase (SOD) activity as well as glutathione (GSH) and malondialdehyde (MDA) levels. The detection method was performed according to the manual descriptions.

### 2.5. Detection of T-Globulin, IgA, IgG, and IgM Content in Serum

The content of T-globulin in serum was detected by an automatic biochemical analyzer (VetScan^®^ VS2, Tuorui, Beijing, China), and the contents of IgA, IgG, and IgM were determined with an enzyme-linked immunosorbent kit (mlbio, Shanghai, China) according to the manual descriptions.

### 2.6. RNA Transcription and Real-Time Fluorescence Quantitative PCR (qPCR)

TRIzol method was used to extract total RNA from spleen tissue and then dissolved in 20 μL RNase-free water. After measurement of the concentration, the total RNA was reversely transcribed into cDNA using a reverse transcription kit (Sangon Biotech Shanghai, China). According to the concentration, the cDNA was diluted to perform the qPCR. qPCR was performed using a qPCR instrument (Bio-Rad, CA, USA). Table 1 displays the primers used in this experiment. The relative mRNA in spleen tissues was calculated by use of 2^−ΔΔCt^ method.

### 2.7. Western Blotting (WB)

RIPA lysate with 1% phenylmethanesulfonyl fluoride (PMSF) was used to extract total protein from spleen tissues. We mixed the protein sample with loading buffer in proportion, and it was centrifuged and boiled. The sequestering of protein samples was achieved by SDS-PAGE, and we transferred them to the polyvinylidene fluoride (PVDF) membrane undergoing 2 h blocking with 5% skimmed milk at 28 ± 2 °C. After PVDF was washed out, we implemented incubation with primary antibody diluent, including β-actin (1:3000; Abmart, Shanghai, China), NF-κB (1:1500; Abmart, Shanghai, China), p-IκBα (1:1000; Abmart, Shanghai, China), TNF-α (1:500; Wanleibio, Shenyang, China), IFN-γ (1:500; Wanleibio, Shenyang, China), COX-2 (1:600; Wanleibio, Shenyang, China), and IL-2 (1:300; Wanleibio, Shenyang, China) for 12 h at 4 °C. Washed with PBST, the PVDF membrane of β-actin was cultured in secondary antibody of goat anti-mouse (1:2500, Bioss, Beijing, China), and the PVDF membrane of NF-κB, p-IκBα, TNF-α, COX-2, IFN-γ, and IL-2 were incubated in secondary antibody of goat anti-rabbit (1:5000, Bioss, Beijing, China) for 1 h at 28 ± 2 °C. The ECL Plus kit (Beyotime, Shanghai, China) was used to visualize the protein bands, and the fully automatic chemiluminescence imaging system was used to photograph the fluorescence of protein bands. ImageJ software (Version 1.38) was applied to perform the quantitative protein analysis.

### 2.8. Data Assessment

Mean ± standard deviation was employed to present all data, and we analyzed the data with Graphpad Prism8 software (GraphPad Software Inc, San Diego, CA, USA) and IBM SPSS Statistics 25 software (IBM, Armonk, NY, USA). The one-way analysis of variance was adopted for revealing statistical difference between any two groups. *P* below 0.05 indicates that the data were statistically significant, and the different lowercase letters on the bar graphs indicate statistical differences between the two groups.

## 3. Results

### 3.1. The Effects of Cr^6+^ and Se on the Spleen Organ Index of Broiler

**Figure 1:**
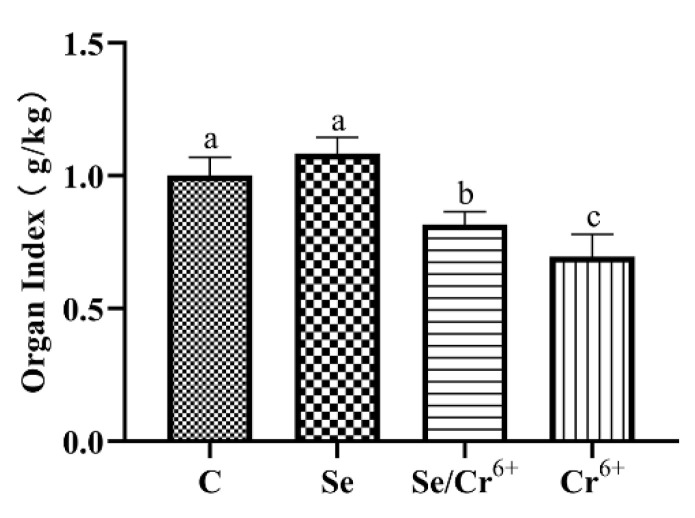
The effects of Cr^6+^ or/and Se on organ index in broiler chicken. Bars with different lowercase letter reflect evident differences (*p* < 0.05). Data are expressed as the means ± SD.

With the aim of judging the protective performance of Se against Cr^6+^-triggered injury to the broiler spleen, we measured the spleen organ index (Figure 1). There was no significant difference between C and Se groups. We observed prominently falling spleen organ indexes in Se/Cr^6+^ and Cr^6+^ groups relative to the C group. However, the Cr^6+^ group exhibited lower spleen organ indexes than Se/Cr^6+^group. These showed that Se could alleviate the damaging effect of Cr^6+^ on the growth performance and spleen tissues of broilers.

### 3.2. The Effects of Cr^6+^ and Se on Oxidative Stress in Broiler Spleen Tissues

**Figure 2:**
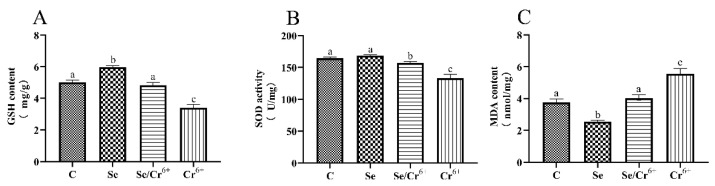
The effects of Cr^6+^ or/and Se on oxidative stress in broiler chicken. Bars with different lowercase letters reflect evident differences (*p* < 0.05). Data are expressed as the means ± SD. (**A**) represents the content of glutathione (GSH); (**B**) represents the activity of superoxide dismutase (SOD); and (**C**) represents the content of malondialdehyde (MDA).

The effects of Cr^6+^ and Se on levels of GSH, SOD, and MDA in spleen tissues are shown in Figure 2. It was unfolded that the content of GSH in the Se group significantly increased in contrast to the C group (*p* < 0.05), and the activity of SOD in the Se group experienced no significant change. Additionally, no difference in the GSH level was uncovered between the Se/Cr^6+^ group and C group, and the activity of SOD showed a downward-sloping trend (*p* < 0.05) in the corresponding groups. The levels of GSH and SOD dropped dramatically in the Cr^6+^ group compared with all other groups. As one of the oxidation products, MDA’s content in the Se group was significantly decreased compared to the C group. The content of MDA in the Cr^6+^ group rose (*p* < 0.05) relative to that in the C group. Additionally, there was no significant change between C and Se/Cr^6+^ groups. However, the content of MDA in the Se/Cr^6+^ group was lower (*p* < 0.05) than that in the Cr^6+^ group. These results suggested that Se could alleviate the oxidative damage caused by Cr^6+^ through increasing GSH and SOD and decreasing MDA in broiler spleens.

### 3.3. The Effects of Cr^6+^ and Se on Immune Function

**Figure 3:**
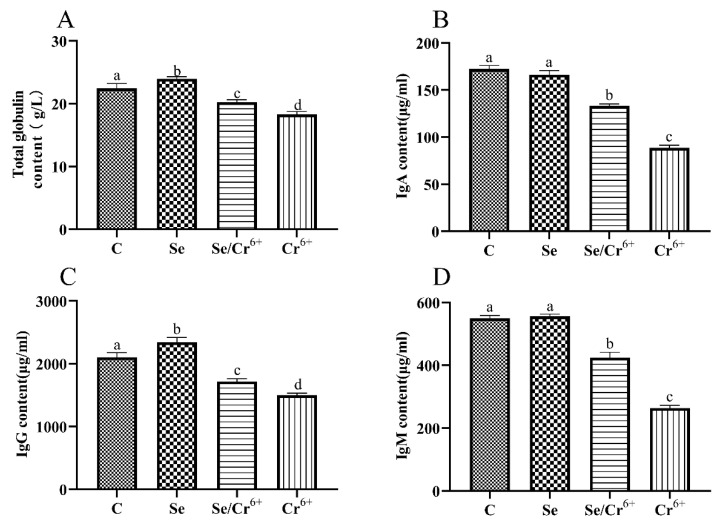
The effects of Cr^6+^ or/and Se on immune indexes in broiler chicken. Bars with different lowercase letters reflect evident differences (*p* < 0.05). Data are expressed as the means ± SD. (**A**) represents the content of total globulin; (**B**) represents the content of immunoglobulin A (IgA); (**C**) represents the content of immunoglobulin G (IgG); and (**D**) represents the content of immunoglobulin M (IgM).

We measured whether Se could alleviate the splenic immune function caused by Cr^6+^ exposure by detecting the contents of T-globulin, IgA, IgG, and IgM in serum (Figure 3). Compared with the C group, the contents of T-globulin and IgG were upregulated (*p* < 0.05) in the Se group, and the contents of IgA and IgM in the Se group had no significant differences. Compared with the C group, the contents of all immunoglobulins and T-globulin in Se/Cr^6+^ and Cr^6+^groups were decreased (*p* < 0.05). However, the contents of all immunoglobulins and T-globulin in the Se/Cr^6+^ group were higher (*p* < 0.05) than that in the Cr^6+^ group. These results suggested that Se can alleviate the immunosuppression caused by Cr^6+^.

### 3.4. The Results of Histopathological Changes Induced by Cr^6+^ and Se

**Figure 4:**
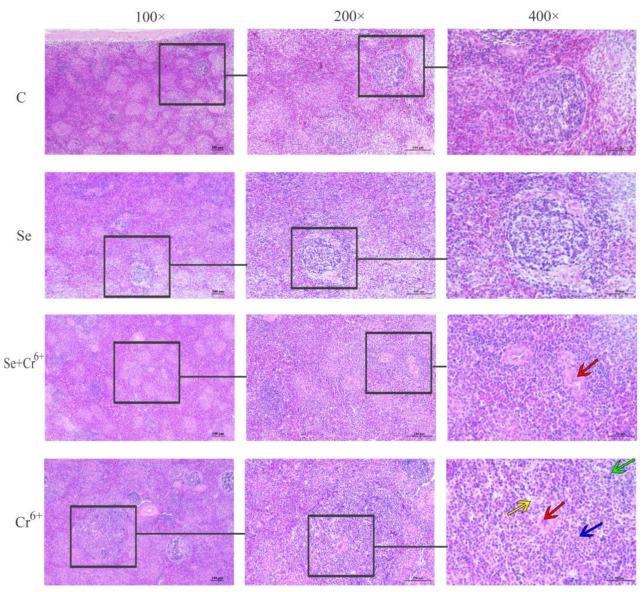
Histopathology observations of spleen tissue. The groups from top to bottom are C, Se, Se/Cr^6+^, and Cr^6+^ groups, and the magnifications from left to right are 100×, 200×, and 400×. Red arrow: aortic thickening; green arrow: inflammatory cell infiltration; yellow and blue arrows: lysis or even dissolution of lymphocyte nucleus.

The observation results of the histology of HE-stained sections of broiler spleens are shown in Figure 4. No obvious histopathological changes were seen in C and Se groups. The results of HE staining showed that Cr^6+^ exposure induced a series of histopathological injuries in broiler spleen tissues, including aortic thickening, the unclear boundary between white and red pulps, inflammatory cell infiltration, and lysis or even dissolution of lymphocyte nucleus, while the above-mentioned symptoms were significantly relieved in the Se/Cr^6+^group. The histopathological observations suggested that Se can effectively alleviate the histopathological damage of spleen tissues caused by Cr^6+^.

### 3.5. The Effects of Cr^6+^ and Se on Inflammatory Cytokines in Broiler Spleens

**Figure 5:**
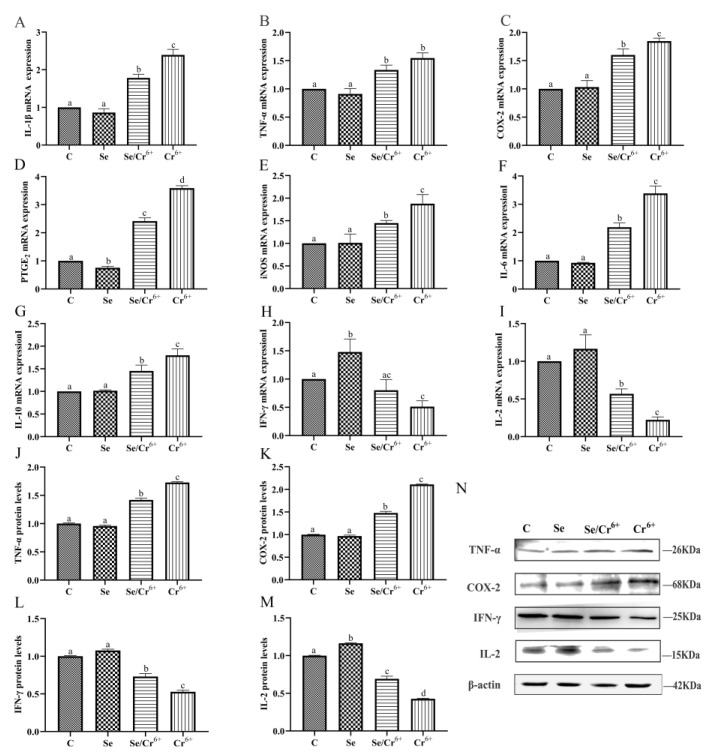
The effect of Cr^6+^ and/or Se on the pro-inflammatory cytokines and immune cytokines in the spleen tissue of broiler. Bars with different lowercase letters reflect evident differences (*p* < 0.05). Data are expressed as the means ± SD. (**A–I**) indicate the mRNA expressions of genes (IL-1β, TNF-α, COX-2, PTGE_2_, iNOS, IL-6, IL-10, IFN-γ and IL-2); (**J–M**) indicate the protein expressions of TNF-α, COX-2, IFN-γ and IL-2; (**N**) indicates the depth of the electrophoresis band by Western blotting for TNF-α, COX-2, IFN-γ and IL-2.

The mRNA and protein expressions of inflammatory cytokines in spleen tissues are shown in Figure 5. The transcription levels of IL-1β, TNF-α, COX-2, PTGE_2_, iNOS, IL-6, IL-10, IFN-γ, and IL-2 mRNAs were detected by qPCR. Compared to the C group, the transcription level of PTGE_2_ slumped (*p* < 0.05), while that of IFN-γ rose (*p* < 0.05) in the Se group. The transcription levels of the other genes had no significant differences between C and Se groups. Compared to the C group, the transcription levels IFN-γ and IL-2 in the Cr^6+^ group were reduced (*p* < 0.05), along with the elevated transcription levels of the other genes (*p* < 0.05) in the corresponding group. The transcription level of IL-2 in the Se/Cr^6+^ group was higher (*p* < 0.05) than that in the Cr^6+^ group, while those of IL-1β, COX-2, PTGE_2_, iNOS, IL-6, and IL-10 were lower in the Se/Cr^6+^ group (*p* < 0.05) than in the Cr^6+^ group.

There were more TNF-α, COX-2, IFN-γ, and IL-2 proteins that were expressed detected by WB. TNF-α, COX-2, and IFN-γ proteins had no significant differences in terms of their expression levels between C and Se groups, but the expression of IL-2 in the Se group rose (*p* < 0.05) relative to the C group. Relative to the C group, the elevation was notable in the protein expression levels of TNF-α and COX-2 in Se/Cr^6+^ and Cr^6+^ groups, but a remarkable reduction was uncovered in the expressions of IFN-γ and IL-2 in Se/Cr^6+^ and Cr^6+^ groups. The Se/Cr^6+^ group demonstrated lower protein expression levels of TNF-α and COX-2 (*p* < 0.05) and higher expressions of IFN-γ and IL-2 (*p* < 0.05) than the Cr^6+^ group.

Thus, expression patterns of inflammation cytokines at mRNA and protein levels were approximately consistent. These results revealed that SeY could alleviate inflammation induced by Cr^6+^ in broiler spleens.

### 3.6. The Functions of Cr^6+^ and Se in the NF-κB Signaling Pathway in Broiler Spleens

**Figure 6:**
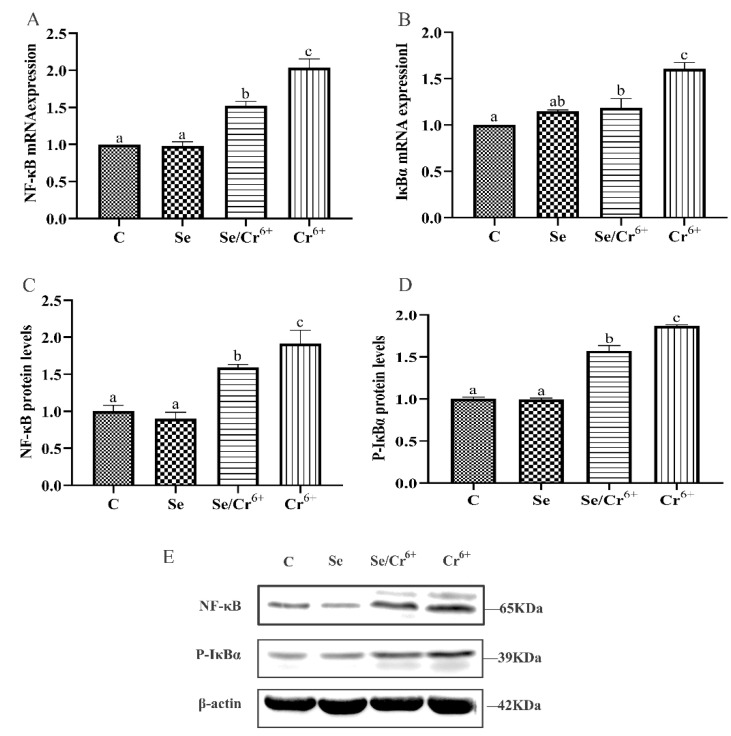
The effect of Cr^6+^ and/or Se on the NF-κB signaling pathway in the spleen tissue of broiler chickens. Bars with different lowercase letters reflect evident differences (*p* < 0.05). Data are expressed as the means ± SD. (**A**,**B**) indicate the mRNA expressions of NF-κB and IκBα; (**C**,**D**) indicate the protein expressions of NF-κB and P-IκBα; (**E**) indicates the depth of the electrophoresis band by Western blotting for NF-κB and P-IκBα.

Figure 6 illustrates the mRNA and protein expressions of NF-κB pathway-associated genes in spleen tissues. The transcription levels of NF-κB and IκBα mRNAs were detected by qPCR, which reflected no remarkable changes in the transcription levels of NF-κB and IκBα between C and Se groups. Nonetheless, they were raised in Se/Cr^6+^ and Cr^6+^ groups (*p* < 0.05) compared to those in the C group. Meanwhile, the transcription levels of NF-κB and IκBα in the Se/Cr^6+^ group were lower (*p* < 0.05) than those in the Cr^6+^ group.

WB validated that C and Se groups did not differ in the expression levels of NF-κB and P-IκBα proteins(*p* > 0.05), but these levels rose in Se/Cr^6+^ and Cr^6+^groups (*p* < 0.05) relative to the C group. Further, they were lower in the Se/Cr^6+^ group (*p* < 0.05) than in the Cr^6+^ group.

Thus, the expression patterns of NF-κB pathway genes at mRNA and protein levels were approximately consistent. These results revealed that SeY could reduce the NF-κB pathway elevated by Cr^6+^ in broiler spleens.

### 3.7. The Results of Bioinformatics Clustering Heat Map in Broiler Spleens by Cr^6+^ and Se Exposure

**Figure 7:**
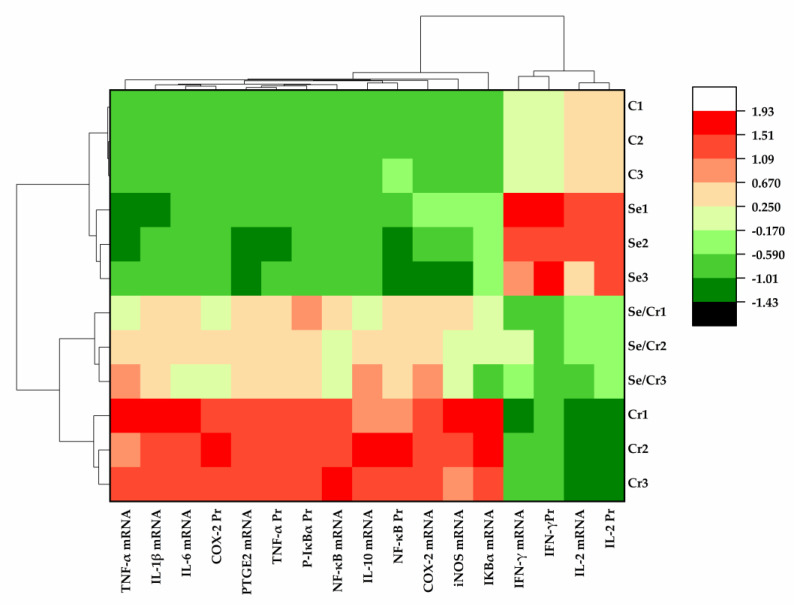
The results of bioinformatics clustering heat map in broiler spleen by Cr^6+^ and Se exposure. C represents the C group, Se represents the Se group; Se/Cr represents the Se/Cr^6+^ group; Cr represents the Cr^6+^ group.

The bioinformatics clustering heat map was used to summarize the previous indicators and each sample (Figure 7). IFN-γ and IL-2 were downregulated by Cr^6+^ exposure, whereas the other pro-inflammatory genes were upregulated. The pro-inflammatory genes in the Se/Cr^6+^ group were effectively reduced compared to those in the Cr^6+^ group, along with the elevated expressions of IFN-γ and IL-2. Similarly, the expression of NF-κB pathway-associated genes was significantly different by Cr^6+^ and/or Se exposure. In addition, there was no statistical change in inflammation and NF-κB-pathway-associated genes between C and Se groups. C and Se groups were classified together, and Se/Cr^6+^ and Cr^6+^ groups were classified together. Meanwhile, compared with the Cr^6+^ group, the Se/Cr^6+^ group was closer to Se and C groups. It could be deduced that Se plays an indispensable role as a protective agent against inflammation via the NF-κB pathway in broiler spleens.

## 4. Discussion

As a consequence of the wide application of Cr^6+^ in the industry recently, the pollution of Cr^6+^ has become more and more serious. A review has reported that Cr^6+^ exposure is harmful to humans and animals by inducing various damages, such as lung cancer, nasal ulcers, allergic reactions, and contact rhinitis [32]. Therefore, it is very necessary to find a substance that can effectively antagonize Cr^6+^. More and more pieces of evidence have shown that Se can antagonize the toxicity of various heavy metals. For example, the Hg content in merganser muscle tissues in Se-deficient areas was significantly higher than that in selenium-enriched areas [33]; Se could alleviate the inflammatory damage effect of cadmium poisoning in the chicken kidney [34] and also could effectively alleviate oxidative stress induced by Cr^6+^ in chicken brain tissues [35]. Therefore, we speculated that SeY could alleviate the damage of broiler spleen tissues caused by Cr^6+^. In this experiment, we found that exposure to Cr^6+^ led the organ index to decrease, and the addition of SeY would alleviate this phenomenon. We also found the histopathology of broiler spleen tissues was changed by Cr^6+^, including aortic thickening, the inconspicuous boundary between white and red pulps, inflammatory cell infiltration, and lysis or even dissolution of lymphocyte nucleus. However, these histopathological damages were alleviated in the Se/Cr^6+^ group. These results showed that SeY could effectively alleviate the damage of broiler spleen tissue caused by Cr^6+^ exposure. We also detected other indicators, including immune function, oxidative stress, inflammation, and the NF-κB signaling pathway, for disclosing the molecular mechanism developed by SeY to relieve the damage of Cr^6+^ in broiler spleen tissues.

The content of serum globulin had a strong correlation with the contents of immunoglobulins [36]. The content of immunoglobulins is an important indicator for immune function, and immunoglobulins are mainly composed of IgA, IgG, and IgM [37]. Luo et al. reported that the decreased contents of IgA, IgG, and IgM in broiler serum induced by fluoride would eventually downregulate the humoral immune function through reducing and/or activating the lymphocyte [21]. These were consistent with our results. In this work, Cr^6+^ could significantly reduce the contents of T-globulin, IgA, IgG, and IgM in serum, which might cause immunosuppression in broilers. Compared with those in the C group, the contents of T-globulin and IgG slightly were raised in the Se group. However, the contents of T-globulin, IgA, IgG, and IgM in serum predominantly rose in the Se/Cr^6+^ group compared to those in the Cr^6+^ group. These results indicated that SeY could effectively alleviate the immunosuppression induced by Cr^6+^ exposure in broilers.

Heavy metals could induce various organs’ toxicity, mainly caused by oxidative stress. For example, Cr^6+^ could cause oxidative stress and further induce cell necrosis in broiler liver tissues [29]; subchronic Pb exposure could lead oxidative stress to trigger apoptosis in mouse spleen tissues [10]; the oxidative stress induced by Cu caused inflammation in the spleen, thymus, and bursa of the fabric of chickens [14]. Moreover, the hepatotoxicity experiments also showed that Cr^6+^ participated in the redox reaction of reducing substance glutathione, and a large amount of ROS was produced, resulting in abnormal glucose and lipid metabolism in vivo and in vitro [38]. Therefore, oxidative stress is one of the important mechanisms for studying the toxicity of Cr^6+^. In this study, the content of MDA rose significantly, and the level of SOD and the content of GSH slumped significantly in spleen tissues after Cr^6+^ exposure. The above data were the same as the result of oxidative damage caused by Cr^6+^ in Chinese lobster [39]. Furthermore, a study found that the content of GSH and the levels of SOD were slightly increased when supplementing appropriate SeY to increase the organism’s antioxidant system in spleen tissues [40]. These indicated that Cr^6+^-induced oxidative damage and SeY could maintain a better antioxidant system in spleen tissues. Simultaneously, compared with the Cr^6+^ group, the content of MDA dramatically slumped, and the level of SOD and the content of GSH rose significantly in the Se/Cr^6+^ group, but these data still did not reach a normal level. These results showed that SeY could effectively alleviate oxidative damage caused by Cr^6+^ in broiler spleens by upregulating the antioxidant system.

Oxidative stress could induce the release of cytokines [41]. Many studies have shown that cytokines are related to inflammatory responses, especially TNF-α and IL-1β, which can mediate many local and systemic inflammatory responses to activate IL-6 and other pro-inflammatory cytokines. TNF-α induces COX-2 and iNOs to produce a large amount of PTGE_2_ and NO, thereby aggravating the inflammatory response [42,43,44]. In this study, notably, the expressions of IL-1β, TNF-α, COX-2, PTGE_2_, iNOs, IL-6, and IL-10 increased in the Cr^6+^ group more than those in the C group, identical to the inflammatory response-associated gene expressions caused by Arsenic (III) in carp [45]. IL-2 promotes the proliferation of immune cells [46]. IFN-γ is produced by T cells and is related to inflammation and immune responses. IFN-γ promotes the secretion of B cells, thereby enhancing the organism’s immunity [47]. As the spleen tissue was damaged by Cr^6+^, the expressions of IL-2 and IFN-γ were decreased significantly, and the addition of SeY could effectively trigger more IL-2 and IFN-γ to be expressed. Liu et al. found that Se supplementation could effectively reduce IL-1β, IL-6, and TNF-α in the thymus and liver of weaned piglets [24]. In this study, IL-2 and IFN-γ in Se/Cr^6+^ group were relieved compared with those in the Cr^6+^ group. These results demonstrated that SeY could antagonize the damage of Cr^6+^ to the spleen by reducing pro-inflammatory cytokines and increasing immune cytokines.

The NF-κB signaling pathway is one of the regulatory mechanisms to regulate inflammatory factors and immune response [48]. NF-κB is mainly composed of P50 and RelA (P65). Under normal conditions, NF-κB binds to the IκB protein family (IκBα/IκBβ/IκBγ/IκBε), which is an inhibitory protein to keep NF-κB in an inactive state [49]. The most common composition of NF-κB is a trimer composed of P50, RelA (P65), and IκB (1:1:1), of which RelA (P65) is indispensable [50]. When NF-κB is activated, IκB is phosphorylated and dissociated from NF-κB, and NF-κB is transferred into the nucleus to bind to the target genes and enhance their expressions [51]. In this study, Cr^6+^ exposure significantly elevated the expressions of NF-κB, IκBα, and P-IκBα in the broiler spleen, but they were reduced in the Se/Cr^6+^ group compared with those in the Cr^6+^ group. These results were consistent with the experiments as follows. Zhang et al. found that SeY antagonizes the inflammatory damage caused by Cd to chicken cardiomyocytes through the NF-κB signaling pathway [52], and Wang et al. showed that the addition of Se could effectively downregulated the high-expression of NF-κB gene in chicken kidney tissue caused by Cd exposure [34]. These results demonstrated that the addition of SeY could effectively inhibit the phosphorylation of IκBα to prevent NF-κB from entering the nucleus and mitigate the inflammatory response. Therefore, we suggested that SeY can alleviate Cr^6+^ toxicity through inhibiting the NF-κB signaling pathway in broiler spleens.

## 5. Conclusions

In summary, the oxidative stress induced by Cr^6+^ triggers NF-κB pathway-driven inflammatory responses in the broiler spleen and further reduces the immune function of broilers. SeY relieved the immunosuppression and inflammation induced by Cr^6+^ exposure by inhibiting the NF-κB signaling pathway-mediated by oxidative stress in broiler spleens. This study enriches the theoretical mechanism of Cr^6+^ toxicity to broiler spleen cells and provides a solution for Cr^6+^ toxicity. Next, further in vitro experiments are needed to determine more complex and deep mechanisms.

## Figures and Tables

**Table 1:** The genes and primers used in this study.

Serial Number	Target Gene	Primer Sequence (5′−3′)
L08165	β-actin	Forward 5′-CCGCTCTATGAAGGCTACGC-3′ Reverse 5′-CTCTCGGCTGTGGTGGTGAA-3′
AY765397.1	TNF-α	Forward 5′-ACACGACAGCCAAGTCAACG-3′ Reverse 5′-GCCCTTCCTGTAACCAGATG-3′
DQ393270	IL-1β	Forward 5′-CACCCGCTCCCAGTCCTT-3′ Reverse 5′-TGGGTGACTCCAGCACGAA-3′
NM_205134.1	NF-κB	Forward 5′-GCAGATAGCCAAGTTCAGGATG-3′ Reverse 5′-TCAACGCAGGACCTAAAGACAT-3′
NM_204961.1	iNOs	Forward 5′-CCTGGGTTTCAGAAGTGGC-3′ Forward 5′-CCTGGAGGTCCTGGAAGAGT-3′
HM584710.1	PTGE2	Forward 5′-CGCATCCTCTGGGTTAGCA-3′ Reverse 5′-GTTCCTGTCATTCGCCTTCTAC-3′
DQ470471	IFN-γ	Forward 5′-AAGTCATAGCGGCACATCAAAC-3′ Reverse 5′-CTGGAATCTCATGTCGTTCATCG-3′
AB302327.1	IL-6	Forward 5′-AGAAGTTCACCGTGTGCGAGAA-3′ Reverse 5′-CTGGAGAGCTTCGTCAGGCATT-3′
NM_001167719.1	COX-2	Forward 5′-TTCCATTGCTGTGTTTGAGGT-3′ Reverse 5′-TGTCCTTTCACTGCTTTCCAT-3′
HE608819	IL-2	Forward 5′-GGAGCATCTCTATCATCAGCAA-3′ Reverse 5′-TGGGTCTCAGTTGGTGTGTA-3′
AJ621254	IL-10	Forward 5′-CCAGCACCAGTCATCAGCAGAG-3′ Reverse 5′-GCAGGTGAAGAAGCGGTGACAG-3′
